# The microbiome alterations of supragingival plaque among adolescents using clear aligners: a metagenomic sequencing analysis

**DOI:** 10.1186/s40510-024-00547-x

**Published:** 2024-12-16

**Authors:** Chunlin Wang, Chao Zhang, Shan He, Qiuyu Wang, Hai Gao

**Affiliations:** 1 Department of Orthodontics, Stomatological Hospital, School of Stomatology, Southern Medical University, Guangzhou, 510280 China; 2 Department of Periodontology and Oral Implantology, Stomatological Hospital, School of Stomatology, Southern Medical University, S366 Jiangnan Boulevard, Haizhu District, Guangzhou, Guangdong 510280 China

**Keywords:** White spot lesions, Clear aligners, Oral microbiome, Virulence factors, Adolescents, Supragingival plaques

## Abstract

**Background:**

White spot lesions (WSLs) may develop in adolescents undergoing clear aligner (CA) therapy with poor oral hygiene. The specific effects of CAs on the microbial composition and functional characteristics of supragingival plaques remain unclear. The present study investigated the shift in the supragingival microbial community induced by CAs in adolescents through metagenomic technology.

**Methods:**

Fifteen adolescents (12–15 years old) with Invisalign appliances were recruited. Supragingival plaque specimens were obtained twice, before treatment (T_1_) and three months after treatment (T_2_). All the bacterial plaque specimens were analyzed for microbial communities and functions using metagenomic analyses.

**Results:**

A total of 2,840,242,722 reads disclosed 180 phyla, 3,975 genera, and 16,497 microbiome species. During the first three months, the microbial community was relatively stable. The genus level revealed a higher relative abundance of *Capnocytophaga*, *Neisseria*, and *Arachnia* in the T_2_ period. Furthermore, the functional analysis suggested that the relative abundances of folate biosynthesis, biotin metabolism and biofilm formation-vibrio cholerae were increased in the T_2_ period compared to the T_1_ period. Finally, virulence factor analysis demonstrated that the relative abundance of genes associated with type IV pili (VF0082) and polar flagella (VF0473) was higher in the T_2_ period than in the T_1_ period.

**Conclusion:**

In adolescents undergoing CA therapy with poor plaque control, caries progresses quickly within three months and noticeable WSLs develop on the tooth surface. Although the microbial community remained relatively steady and CA therapy did not cause significant changes in the overall functional gene composition in the first three months, virulence factors, including type IV pili and flagella, were more abundant and actively contributed to microorganism adhesion and biofilm formation.

## Introduction

Clear aligners (CAs) are removable and full-coverage orthodontic appliances that patients wear for over 20 h daily. Because of their removable nature, most orthodontists believe that CAs are more suitable for maintaining oral hygiene compared with fixed appliances [1]. However, recent research reported that the use of CAs in adolescents can easily lead to enamel demineralization and the development of white spot lesions (WSLs) [2]. WSLs are characterized by a milky white and opaque appearance of smooth surface enamel, indicating an early stage of dental caries [3]. Recently, Liu and Song [4] assessed the occurrence and severity of WSLs by evaluating intraoral photographs of adolescents before and after the use of CAs. Approximately 35.5% of the individuals developed WSLs during the early stage of CA treatment. Clinical observation suggests a rapid progression of caries among adolescents wearing CAs, and WSLs can become noticeable on the tooth’s surface within one month [5]. Song et al. suggested that CAs may cause an imbalance in oral microecological conditions by altering the abundance, distribution, and metabolic pathway of the oral microbiota during the early stage of treatment [6].

Next-generation sequencing studies often use marker gene analysis, targeting specific genes like 16 S rRNA with conserved primers. This method, known as amplicon-based sequencing or metabarcoding, reveals the diversity and relative abundance of taxa in communities [7]. 16 S rRNA gene sequencing were also used to identify microbiome changes caused by CAs treatment [8–10]. However, these studies mainly focused on the inner surface of the CAs [10] or subgingival plaque [11] and saliva [12]. Few studies have directly investigated the association of supragingival plaque and caries lesions in individuals that use CAs. Moreover, 16 S rRNA gene sequencing is incapable of providing certain key microbiome traits, such as species level and functional profile, which are likely important for understanding the pathogenesis of dental caries [13, 14]. To further clarify changes in the bacterial community and functional genes after wearing CAs for three months. Next-generation sequencing (NGS) technologies were chosen, including whole genome sequencing (WGS) and metagenomics sequencing. Whole genome sequencing (WGS) can elucidate individual microbiological isolates’ antimicrobial resistance and virulence potential during diagnostic procedures. Conversely, metagenomic sequencing facilitates the examination of DNA fragments from multiple microorganisms within a community [15]. Consequently, this study utilized metagenomic sequencing analyses to screen microbial markers, key functional genes associated with dental caries, and changes in the bacterial community and functional genes after wearing CAs for three months [16].

In the present study, we used sterile swabs to extract samples of supragingival plaque from adolescents before and after three months of treatment with CAs. The samples were analyzed by metagenomic sequencing. We also investigated the possible changes in the microbial community, microbiome function, and virulence factors at the early stage of CA treatment.

## Materials and methods

### Study participants

The study was approved by the Research Ethics Committee at the Stomatological Hospital of Southern Medical University (Ref No: EC-CT-[2022]23). This study followed the STROBE guidelines. Study participants (*n* = 15) were recruited from the Department of Orthodontics at the Stomatological Hospital of Southern Medical University and met the following inclusion criteria: (a) age from 12 to 15 years; (b) individuals with permanent dentition and mild to moderate crowding; (c) good oral health without dental caries, periodontal disease, mucosal disease, restorations, and systemic disease; and (d) no history of antibiotics or hormone consumption in the last month. The guardians of the participants signed an informed consent form for participation in the study.

After a clinical oral examination and oral scans, each subject was treated using custom aligners and provided with oral hygiene instructions during the treatment period. At the start of the orthodontic treatment (T_1_), a qualified and fully-trained dentist collected supragingival plaque specimens from the cervical regions of the incisors and first molars across all four quadrants by gently scraping with a sterile cotton swab [17]. Plaque specimens were immediately placed in 1.5-mL Eppendorf tubes and stored at -80 °C until further processing. The follow-up appointments were scheduled at three months (T_2_). A questionnaire was used to gather data regarding the sociodemographic characteristics and dietary and oral hygiene habits of participants at T_1_ and T_2_. Two experienced orthodontists independently evaluated WSLs in pretreatment and post-treatment intraoral photographs with computer monitors in a dark room [6]. The plaque index (PI) was evaluated by visualizing the accumulated plaque and graded according to the modified Loe index [18].

### DNA extraction and metagenomic sequencing

DNA was extracted from supragingival plaque specimens using the E.Z.N.A.^®^ Soil DNA Kit (Omega Bio-tek, Norcross, GA, USA) following the manufacturer’s guidelines. The concentration and purity of extracted DNA was determined by TBS-380 and NanoDrop2000, respectively, and agarose gel (1%) was used to determine the quality of extracted DNA. To construct a paired-end library, the extracted DNA was fragmented (average size ~ 400 base pairs) by Covaris M220 (Gene Company Limited, China). The paired-end library was constructed using NEXTFLEX Rapid DNA-Seq (Bioo Scientific, Austin, TX, USA). The blunt end of the fragments received adapters with a complete complement of sequencing primer hybridization sites. Paired-end sequencing was performed using Illumina Hiseq Xten (Illumina Inc, San Diego, CA, USA) and NovaSeq Reagent Kits/HiSeq X Reagent Kits at Majorbio Bio-Pharm Technology Co, Ltd. (Shanghai, China) following the manufacturer’s guidelines (www.illumina.com). The sequencing data of this study have been deposited to the NCBIShort Read Archive database.

### Sequence quality control and genome assembly

The sequencing data were analyzed using the Majorbio Cloud free online platform (www.majorbio.com). Fastp software (https://github.com/OpenGene/fastp, version 0.20.0) was used to remove the low-quality reads (with quality value < 20, length < 50 bp or having N-bases) after trimming the paired-end Illumina reads [19]. After aligning the human genome by BWA (version 0.7.9a) [20] (http://bio-bwa.sourceforge.net), hits related to reads and their mated reads were eliminated. The MEGAHIT software (version 1.1.2) [21] (https://github.com/voutcn/megahit) along with succinct de Bruijn graphs was used to assemble the metagenomics data. Final assembling result included contigs of 300 bp or longer, which were used for gene prediction and annotation analysis.

### Gene prediction and functional annotation

This study used Prodigal [22]/MetaGene [23] (http://metagene.cb.k.u-tokyo.ac.jp/) to determine open reading frames (ORFs) for each assembled contig. We used the NCBI translation table (http://www.ncbi.nlm.nih.gov/Taxonomy/taxonomyhome.html/index.cgi?chapter=tgencodes#SG1) to predict ORFs longer than 100 bp, which were recovered and translated into amino acid sequences. The CD-HIT software (version 4.6.1) [24] (http://www.bioinformatics.org/cd-hit/) was used to construct a non-redundant gene catalogue with the alignment of 90% sequence identity and coverage. SOAPaligner (version 2.21) [25] (http://soap.genomics.org.cn/) was used to align high-quality reads with non-redundant gene catalogues and calculate gene abundance with a 95% identity. For taxonomic annotations, we used Diamond software (version 0.8.35) [26] (http://www.diamondsearch.org/index.php) to align representative sequences of the non-redundant gene catalogue with the NR database (a cutoff e-value of 1e-5).

Kyoto Encyclopedia of Genes and Genomes (KEGG) annotation was performed using Diamond against the KEGG database (http://www.genome.jp/keeg/) with an e-value cutoff of 1e-5. Virulent factor annotation was performed by Diamond against the Virulence Factor Database (VFDB) database (http://www.mgc.ac.cn/VFs/) at a cutoff e-value of 1e-5.

### Data visual exhibition, statistical, and Bioinformatics Analysis

Continuous variables were presented as mean ± standard deviation for normally distributed data, and as median with interquartile range for non-normally distributed data. Categorical variables were reported as percentages. Normality of distribution was assessed using the one-way ANOVA test, while non-normal distribution was evaluated using the Kruskal–Wallis test, followed by paired t-tests for pairwise comparisons. All statistical analyses were conducted using R software packages or SPSS version 21.0 (IBM). Characterized by the integration of non-parametric testing and biological significance, LEfSe is a robust tool for identifying biomarkers from microbial metagenome data [27]. Consequently, LEfSe analysis was conducted to evaluate the robustness of the classifier constructed using the random forest method. Here, bacterial species with LDA score > 2 and *P* < 0.05 were considered to be signifcant. The alpha diversity of the samples was assessed based on the Chao1, ACE, Shannon, and Simpson indices at the species level. Comparisons of alpha diversity between the two groups were performed with the Wilcoxon rank sum test. The beta diversity of the samples was assessed by Principal Component Analysis (PCA) based on Euclidean distance, principal coordinate analysis (PCoA) based on Bray–Curtis distance, and Nonmetric multidimensional scaling (NMDS) ordination based on Bray–Curtis distances according to the abundance tables of microbial taxa at the genus level.

## Results

### Characteristics of the study population

The general and demographic characteristics of the participants are presented in Table 1. After wearing CAs, the oral hygiene habits of participants become much better, including frequently eating sweet snacks, consuming sugary foods, teeth brushing, and brushing duration each time. All these factors contributed to an increase in the number of adolescents with plaque indexes above 2–3 grades and with more than two caries lesions (Table 1).

**Table 1:** Characteristics of study participants (*n* = 15)

Characteristic	T_1_ *n* (%)	T_2_ *n* (%)
Age (y)	13.6 ± 2.6	
Sex		
Female	9 (60)	
Male	6 (40)	
White spot lesions		
≤1	10 (67)	6 (40)
≥ 2	5 (33)	9 (60)
Plaque index		
≤ 1	9 (60)	3 (20)
2–3	6 (40)	12 (80)
Frequency of sweet drink intake		
1–2 times/d	5 (33)	3 (20)
≥ 3 times/d	10 (67)	12 (80)
Frequency of snack intake		
1–2 times/d	6 (40)	4 (27)
≥ 3 times/d	9 (60)	11 (73)
Frequency of eating while wearing CAs		
≤ 1 time/d	15 (100)	12 (80)
≥ 2 times/d	0 (0)	3 (20)
Times of teeth brushing		
< 2 times/d	9 (60)	4 (27)
≥ 2 times/d	6 (40)	11 (73)
Brushing duration each time		
≤ 2 min	11(73)	5 (33)
> 2 min	4 (27)	10 (67)
Use of mouthwash		
Yes	4 (27)	12 (80)
No	11 (73)	3 (20)
Hours of wearing CAs		
≤ 15 h/d	15 (100)	11 (73)
> 15 h/d	0 (0)	4 (27)

T_1_, before treatment; T_2_, three months after treatment

### Sample collection, sequencing, and quality control

A total of 40 plaque specimens from 15 adolescents obtained at T_1_ and T_2_ were analyzed, which yielded 2,840,242,722 (428.87GB) high-quality sequence reads after the removal of host contaminants and low-quality data. The analysis identified a total of 16,497 species, 3,975 genera, and 180 phyla.

### Microbial characterization

**Fig. 1:**
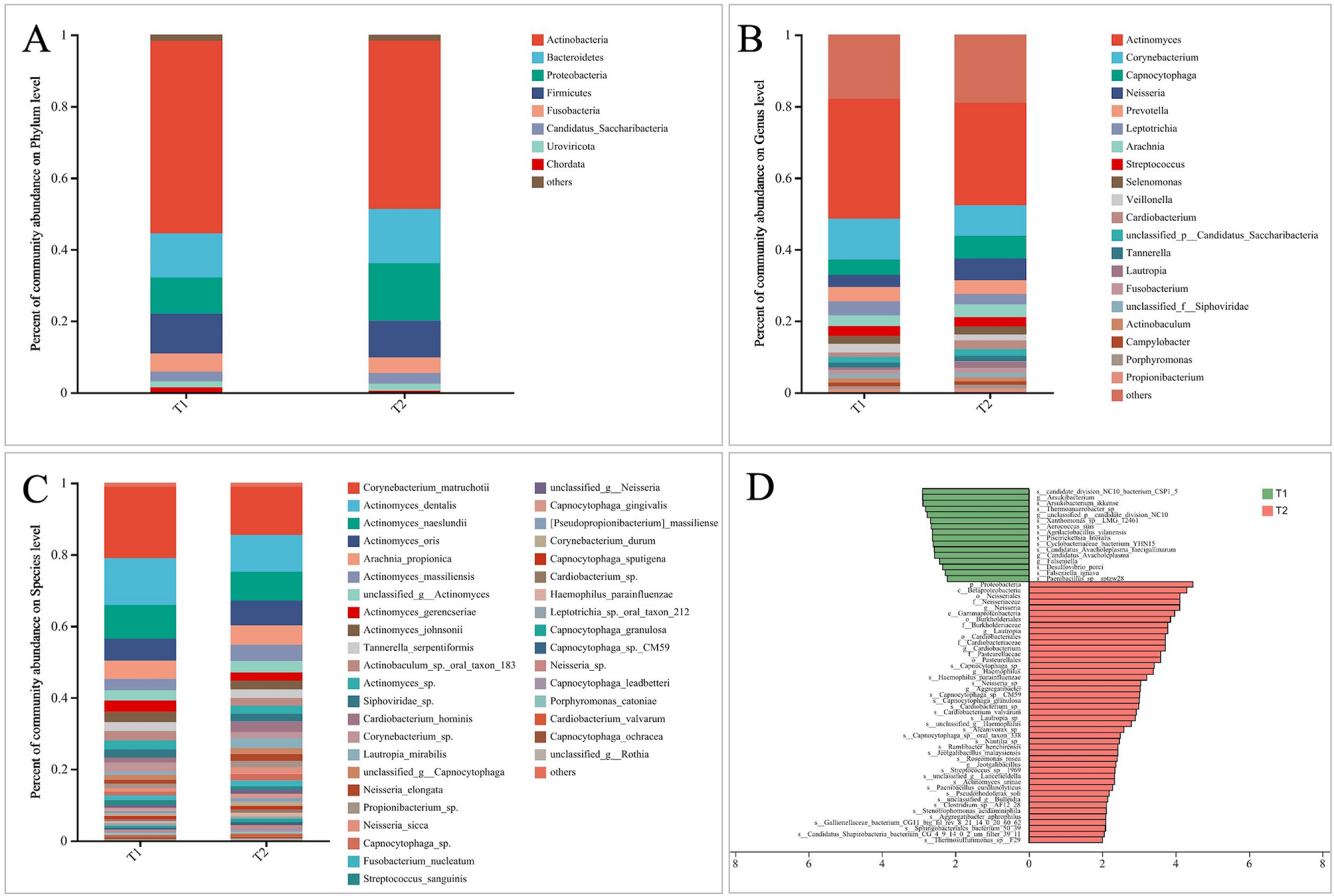
Relative abundances of plaque microbiota in two periods. (**A**–**C**) Relative abundances of phyla, genera, and species from the annotated microbial taxa are shown in a bar plot. **D**. Significant differences in the relative abundance of plaque microbial taxa between the two periods, as identified by linear discriminant analysis (LDA) effect size analysis (LEFSe) (DUNN test, *p* < 0.05)

We investigated and compared the relative abundances of microbial communities at various levels. At the phylum level, *Actinobacteria*, *Bacteroidetes*, *Proteobacteria*, *Firmicutes*, and *Fusobacteria* were the most abundant taxa in the two periods. Three months of treatment enhanced the abundance of *Proteobacteria* and *Bacteroidetes* and diminished the abundance of *Actinobacteria* and *Firmicutes* (Fig. 1A). At the genus level, *Actinomyces*, *Capnocytophaga*, *Corynebacterium*, *Prevotella*, *Neisseria*, *Leptotrichia*, *Arachnia*, *Streptococcus*, *Veillonella*, *Selenomonas*, and *Cradiobacterium* were the most dominant phylotypes at both periods (Fig. 1B). *Actinomyces*, *Corynebacterium*, and *Leptotrichia* were more abundant before treatment, and *Capnocytophaga*, *Neisseria*, and *Arachnia* became more abundant after three months of treatment (Fig. 1B). At the species level, *Actinomyces-dentalis*, *Corynebacterium-Matruchotii*, *Actinomyces-naeslundii*, *Actinomyces-oris*, and *Arahchnia-propionica* comprised a major fraction of the total microbial abundance in both periods, which suggests an association of these species with the stable plaque microflora. Furthermore, *Corynebacterium-matruchotii* and *Actinomyces-dentalis* were more abundant before treatment, while *Actinomyces-oris*, *Arahchnia-propionica*, and *Actinomyces-massiliensis* were enriched after three months of treatment (Fig. 1C).

The time-dependent alteration in taxonomic composition was further characterized using the LEfSe test method (Fig. 1D), which identified the presence of distinct oral microbiome taxa during both periods. Sixteen taxa, including candidate division NC10 bacterium CSP and Arsuklbacterium ikkense, were in high abundance before treatment. In contrast, 45 taxa had higher abundances after treatment, including *Proteobacteria*, *Betaproteobacteria*, *Neisseriales*, *Neisseriaceae*, *Neisseria*, *Gammaproteobacteria*, *burkholderiales*, and *burkholderiaceae*. The differential effect was significantly influenced by the abundance of these species.

**Fig. 2:**
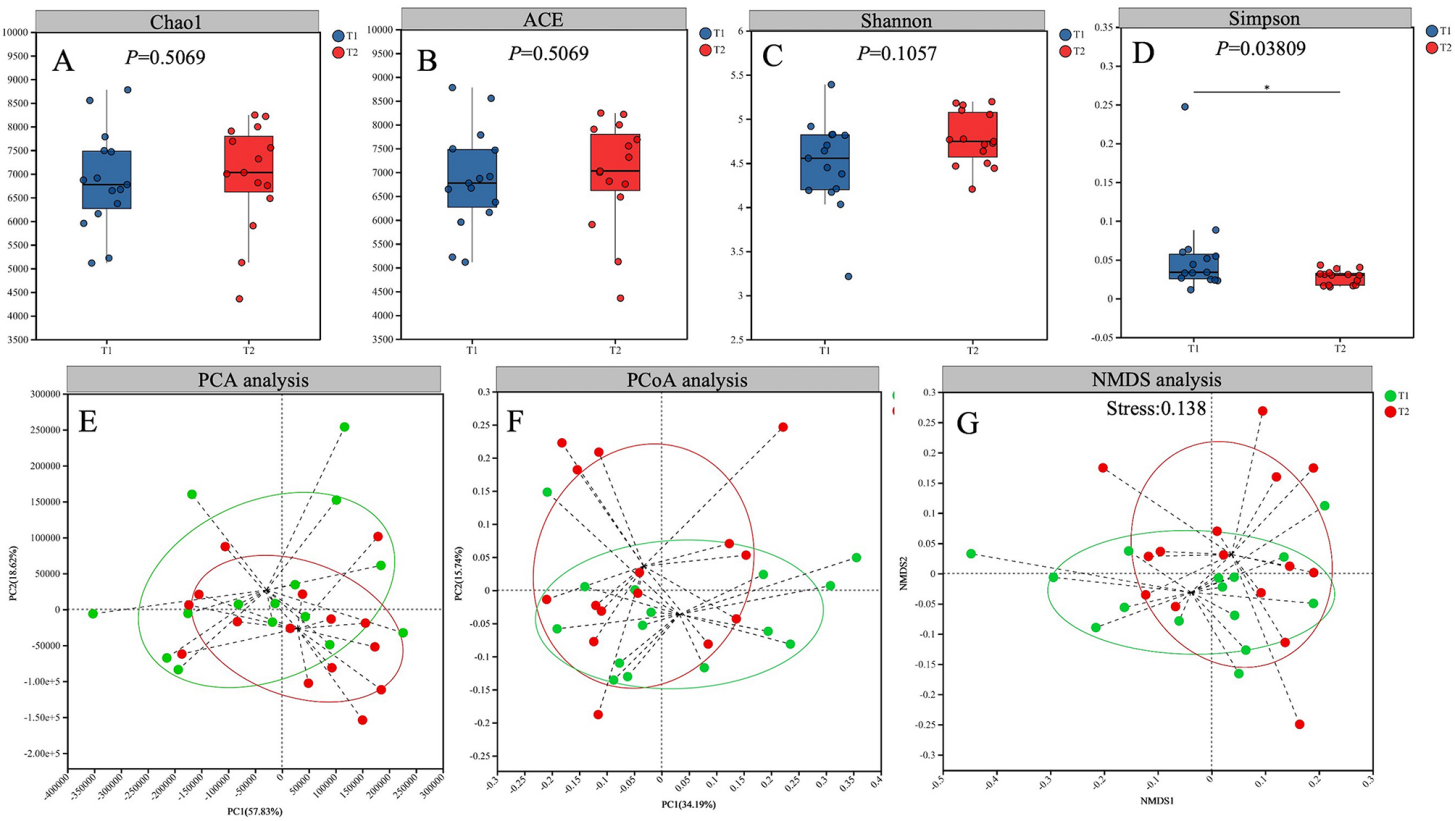
Microbial diversity analysis. Box plots of alpha diversity at the species level (**A**) Chao1; (**B**) ACE; (**C**) Shannon; (**D**) Simpson (*p < 0.05, wilcoxon). (**E**) Principal Component Analysis (PCA) based on Euclidean distance at the genus level. (**F**) Principal coordinate analysis (PCoA) based on Bray–Curtis distance at the genus level. (**G**) Nonmetric multidimensional scaling (NMDS) ordination based on Bray–Curtis distances according to the abundance tables of microbial taxa at the genus level. Each data point represents an individual sample. Circles in different colors represent different groups

**Fig. 3:**
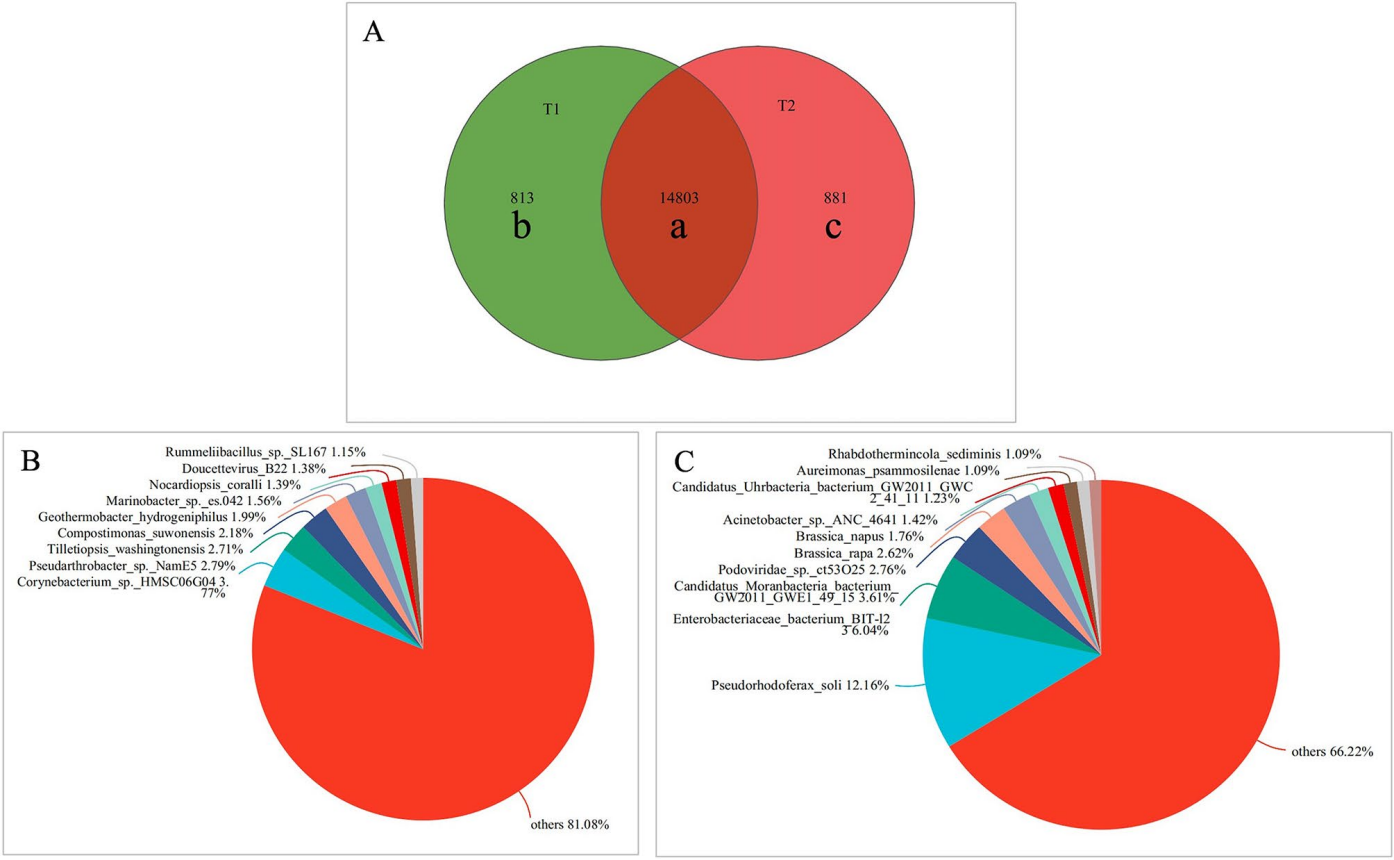
(**A**) Venn diagram at the species level.The overlap region a represents the microbiome shared in all samples between the two period. Region b represents the unique taxa before treatment and Region c represents the unique taxa after treatment. (**B**) Microbiome pieplot at the species level (T1 only). (**C**) Microbiome pieplot at the species level (T2 only)

The results of alpha diversity analysis showed that the Simpson indices in T_2_ group were significantly lower than those in the T_1_ group (*P* < 0.05)(Fig. 2D), and the Chao1, ACE, and Shannon indices were not significantly different between the two groups (Fig. 2A-C). To compare the two periods in terms of plaque microbiome diversity, we reported the Principal Component Analysis (PCA) (Fig. 2E), principal coordinate analysis (PCoA) (Fig. 2F) and nonmetric multidimensional (NMDS) scaling analysis (Fig. 2G). The findings demonstrated that the samples from two times overlapped; however there was a certain tendency of separation between the two periods. The core microbiome, defined as the group of samples shared before and after treatment, was depicted through a Venn diagram. Figure 3A displays the results detected using the Venn diagram at the species level. The present study identified 16,497 species. The overlap region a (14,803 species) represents the microbiome shared in all samples from two periods. Region b (813 species) and region c (881 species) corresponded to the unique taxa before and after treatment, respectively. Figure 3B illustrates that 813 species are exclusively found in the T1 groups. Specifically, *Corynebacterium_sp._HMSC06G04* and *Pseudarthrobacter_sp._NamE5* constitute 6.56% of Region b, both of which belong to the phylum *Actinobacteria* and the class *Actinomycetia*. Conversely, Fig. 3C demonstrates that 881 species are unique to the T2 groups. Notably, *Pseudorhodoferax_soli* and *Enterobacteriaceae_bacterium_BIT-l23* account for 18.16% of Region c, and both are classified under the phylum *Proteobacteria*.

### Functional characteristics of microbiota

**Fig. 4:**
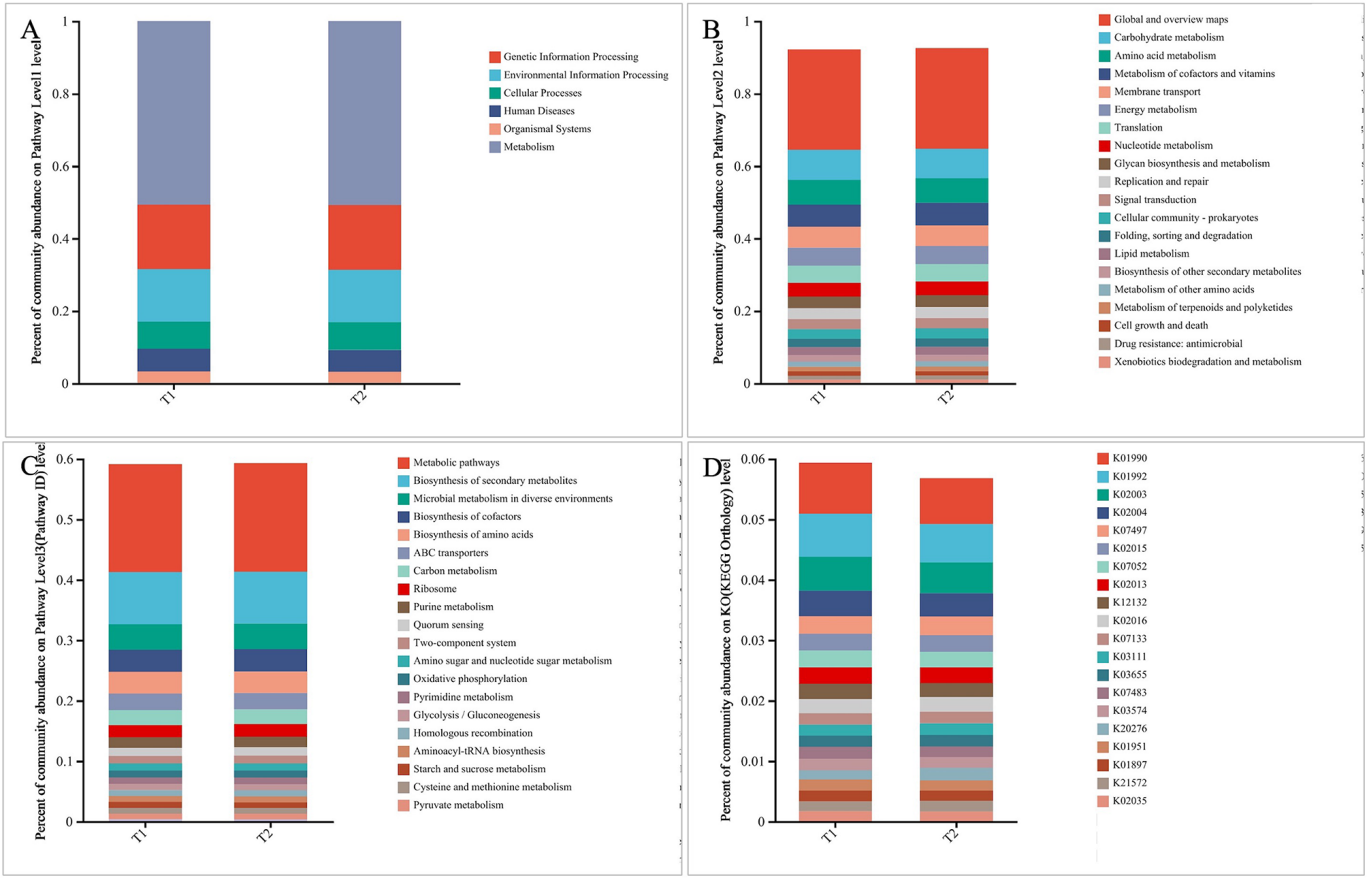
Comparison of the functional characteristics of the microbiome before and after treatment. (**A**) The comparison of functional KEGG between the two groups at the level 1 pathway, (**B**) level 2 pathway (top 20), (**C**) level 3 pathway (top 20), and (**D**) ortholog level (top 20). The horizontal axis represents the relative abundance of annotated genes

KEGG analysis was used to analyze the functional characteristics of oral microbiota of two periods and annotate oral gene directory (Fig. 4A–D). These findings identified six main pathways in KEGG, of which metabolic pathways were the most abundant. In addition, the level 1 pathway in the two periods showed no significant differences (Fig. 4A). The KEGG results showed a comparison of functional KEGG between the two periods in the level 2 pathway, with global and overview maps being the most abundant (Fig. 4B). Furthermore, amino acid, carbohydrate, and cofactor metabolisms were common at level 2 pathways. As shown in Fig. 4C, the level 3 pathway in two periods mainly referred to metabolic pathways, biosynthesis of secondary metabolites, microbial metabolism in diverse environments, and biosynthesis of amino acids.

**Fig. 5:**
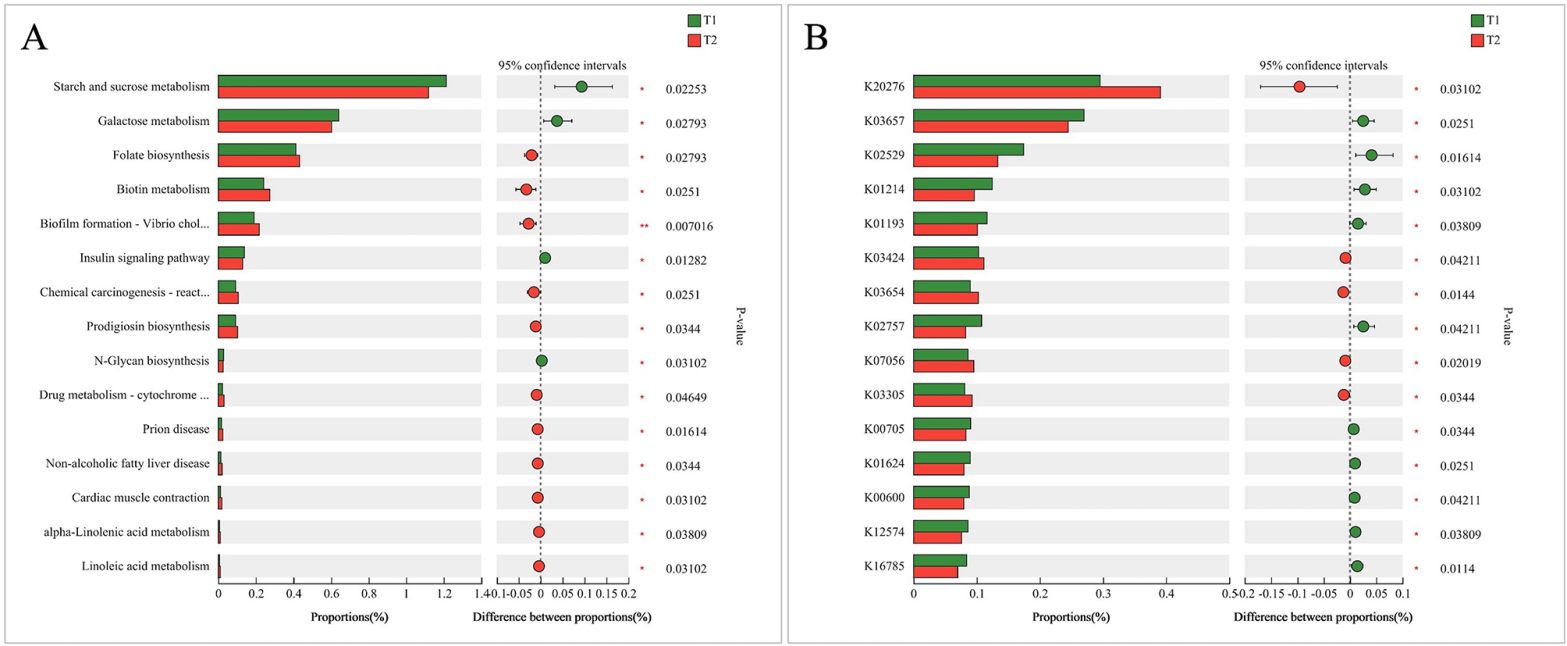
Imputed metagenomic differences between the two periods. The relative abundances of metabolic pathways encoded in each imputed sample metagenome were analyzed in level 3 pathways (**A**) and KOs (**B**)

The relative abundances of metabolic pathways in level 3 pathways of two periods are shown in Fig. 5A–C. The Wilcoxon rank-sum test demonstrated relative abundances of metabolic pathways encoded in each imputed specimen metagenome (Fig. 5A). The relative abundances of starch and sucrose metabolism, galactose metabolism, and insulin signaling pathway significantly decreased after three months of treatment. The relative abundances of folate biosynthesis, biotin metabolism, biofilm formation–vibrio cholerae, and chemical carcinogenesis–reactive oxygen species were significantly increased in the T_2_ period. The Wilcoxon rank-sum test also demonstrated differences in KEGG Orthology (KO) levels between two periods (Fig. 5B). These results indicated that K20276, K03424, K03654, K07056, and K03305 in the T_2_ period were significantly greater than those in the T_1_ period, while K03657, K02529, K01214, and K01193 were significantly lower in the T_2_ period compared with the T_1_ period.

**Fig. 6:**
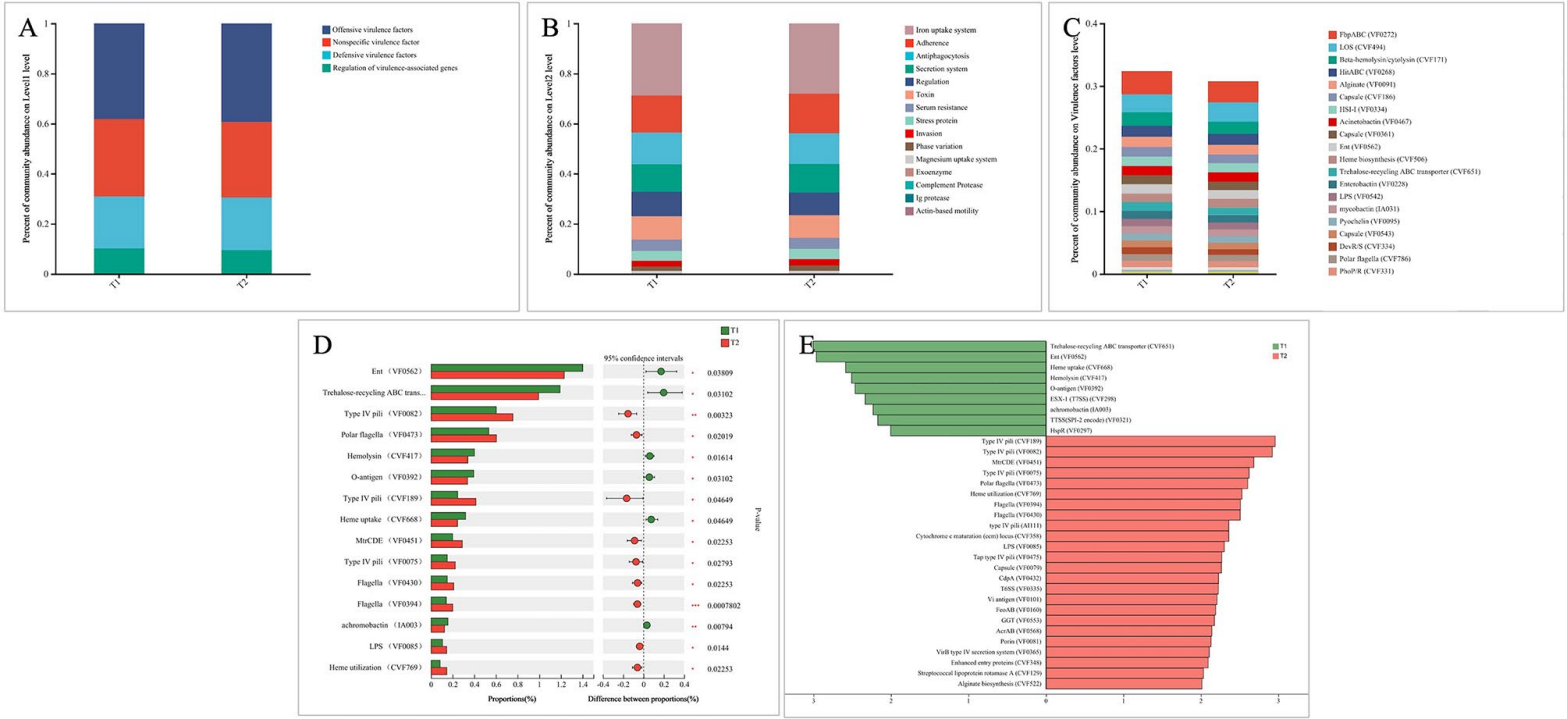
Comparison of the functional characteristics of the microbiome before treatment and after treatment. (**A**) The comparison of functional VFDB between the two groups at the level 1 pathway, (**B**) level 2 pathway, and (**C**) virulence factors (top 20). The horizontal axis represents the relative abundance of annotated genes. The relative abundances of virulence factors in each imputed sample metagenome were analyzed. (**D**) Linear discriminant analysis (LDA) effect size analysis (LEFSe) was used to identify VFs (**E**) with significant differences in relative abundance between the two periods

The virulence factors (VFs) at level 1 are generally graded into four key types, which include offensive virulence factors, nonspecific virulence factors, defensive virulence factors, and regulation of virulence-associated genes (Fig. 6A). Moreover, at level 2, the VFs key types were categorized into various subtypes (level 2), which include iron uptake system, adherence, antiphagocytosis, secretion system, regulation, toxin, serum resistance, stress protein, invasion, and phase variation (Fig. 6B). Figure 6C shows that the virulence factors in the two periods mainly involved fbpABC, LOS, and Beta hemolysin/cytolysin. The results of Wilcoxon rank-sum test for the relative abundances of virulence factors suggested that the relative abundances of Ent (VF0562), trehalose-recycling ABC transporter (CVF651), hemolysin (CVF417), and O-antigen (VF0392) in the T_1_ period were significantly greater than in the T_2_ period (Fig. 6D). However, the relative abundances of type IV pili (VF0082), polar flagella (VF0473), and type IV pili (CVF189) in the T_2_ period were significantly augmented compared with those in the T_1_ period. Figure 6E shows the virulence factors between two periods by LEFSe. The relative abundances of trehalose-recycling ABC transporter (CVF651), Ent (VF0562), Heme uptake (CVF668), and Hemolysin (CVF417) in the T_1_ period were significantly decreased (Fig. 6E). Type IV pili (CVF189), type IV pili (VF0082), mtrCDE (VF0451), and type IV pili (VF0075) increased after three months of treatment with CAs.

**Fig. 7:**
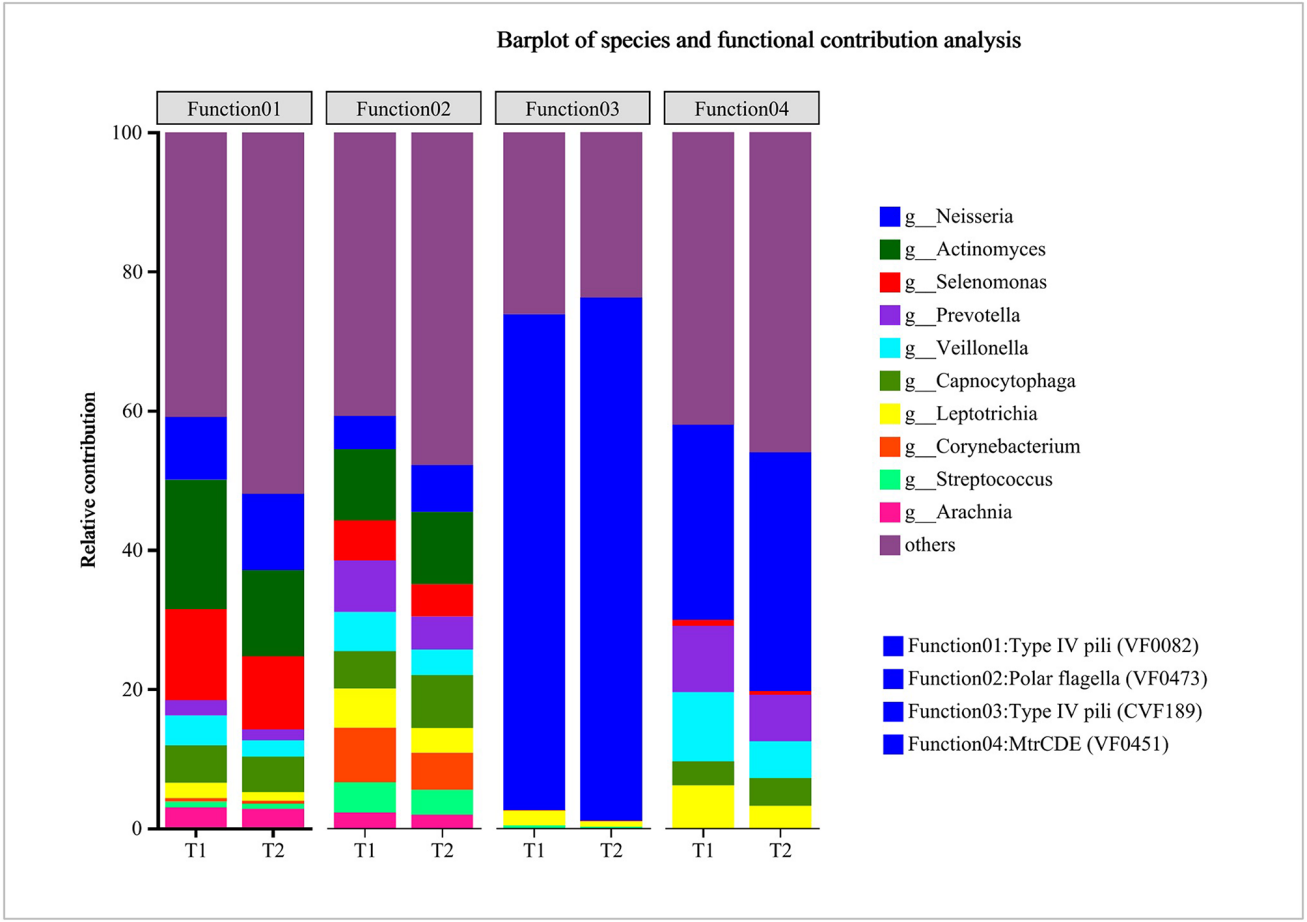
Genus (top 10) contributions to key functions. For each identified virulence factor, HUManN2 was used to calculate the species contribution and showing genus (top 10) contributions to four factors correlated to dental caries

HUManN2 was used to determine the contribution of different species to VFs. Figure 7 presents a bar plot of the species and functional contribution analysis. We chose four VFs, including type IV pili (VF0082), polar flagella (VF0473), type IV pili (CVF189), and mtrCDE (VF0451), which were significantly increased in the T_2_ period. For type IV pili (VF0082), *Neisseria*, *Actinomyces*, *Selenomonas*, and *Capnocytophaga* were the main contributing species in the two periods, and the contribution of *Neisseria* for these VFs increased following treatment. For polar flagella (VF0473), *Neisseria*, *Actinomyces*, and *Selenomonas* are the main contribution genera in two periods. The contribution of *Neisseria* for these factors also increased after treatment. For type IV pili (CVF189) and mtrCDE (VF0451), *Neisseria* was the main contributing genus in the two periods.

## Discussion

The present study investigated the effect of using CAs on the supragingival microbial community in adolescents. Supragingival plaque specimens from CA-using adolescents were analyzed using metagenomic technology for shifts in microbial communities. The demand for CAs has increased over the past decades due to patients’ desire to have less noticeable and more comfortable orthodontic appliances. However, the use of CAs for a prolonged time may compromise the patient’s oral hygiene by diminishing the self-cleaning ability of teeth and reducing the cleaning and flushing action of the saliva and tongue. Developing poor oral hygiene may facilitate the proliferation of pathogenic microbial species and shift physiological oral flora [8, 10, 28]. Albhaisi et al. investigated the association of CA therapy and WSL development using a higher sensitivity system (quantitative light-induced fluorescence images) and demonstrated that patients wearing CAs for three months developed large and shallow WSLs on tooth surfaces [2]. Our findings are in accordance with those of Albhaisi et al. [1], who demonstrated that caries progress rapidly in adolescents with CAs. In the present study, the development of WSLs was noticeable on the tooth surface (especially on lateral incisors) after wearing CAs for 3 months. This may be related to two reasons: first, young permanent teeth are more susceptible to caries due to lower levels of mineralization in the surface enamel. The second is the poor awareness of oral hygiene maintenance and compliance in adolescents [4].

The plaque index results showed that the adolescents’ oral hygiene deteriorated even with guidance on oral hygiene maintenance. Yan et al. reported a lowering of pH in align-users with time [10]. The dysbiosis of the bacterial plaque attached to teeth is the key etiological factor in the development of caries lesions [13, 29, 30]. Acidogenic bacteria, particularly *Streptococcus mutans* and *Lactobacilli*, can metabolize carbohydrates to produce acids and decrease the pH of plaque, leading to the development of caries lesions [31]. However, a gap remained in the understanding of the apparent shift in the microbial community caused by CA treatment since these individual species were only a part of complex communities. Therefore, this study screened microbial markers and key functional genes associated with dental caries and changes in the bacterial community and functional genes over time. In this study, the overall taxonomic compositions were similar, and most of the species were shared in the two periods, indicating a comparatively stable bacterial community structure during treatment [32]. Our findings backed the ‘Ecological Plaque Hypothesis,’ which described dental caries as an outcome of a disturbance in the homeostasis of resident microbiota instead of the metabolic activity of certain microorganisms [32].

Although the two periods exhibited similar overall taxonomic compositions, *Bacteroidetes* and *Proteobacteria* abundance at the phylum level, *Capnocytophaga*, *Neisseria* and *Arachnia* at the genus level, and *Actinomyces oris*,* Arachnia propionica*,* and Actinomyces massiliensis* at the species level were more enriched three months after the treatment. *Capnocytophaga* is involved in the formation of bacterial plaque [33] and can assist in predicting childhood caries at an early stage [34]. Johansson et al. [35] also reported that *Prevotella*, *Actinomyces*, and *Capnocytophaga* present in the supragingival plaque microbiota are associated with cariogenic activity in adolescents. Gram-positive aerobes and facultative anaerobes such as *Actinomyces* species and *Streptococci* are primary colonizers. These can be detected in the initial stages of plaque development and play a key role in biofilm formation via coaggregation interactions [36]. *Neisseria* are involved in the metabolism of vitamin B6, nucleotides, and carbohydrate and are significantly linked to dental caries [17].

To investigate the plaque microbiota, we characterized the structural and functional characteristics of microbiota. We observed a similar functional gene composition of the two periods. The carbohydrate and amino acid metabolism were enriched during CA treatment. These findings are in agreement with those of Song et al., who found the association of predominant amino acid metabolism in WSLs with CAs [6]. Cariogenic bacteria generate organic acids and extracellular polysaccharides by metabolizing carbohydrates, which is one of the leading factors for developing dental caries [37]. However, the genes related to carbohydrate metabolism did not present higher relative abundance levels in individuals wearing CAs for three months. This may be attributed to the short time of wearing CAs, which has not caused changes in plaque metabolism. In contrast, the genes associated with folate biosynthesis, biotin metabolism, biofilm formation, *vibrio cholerae*, and chemical carcinogenesis–reactive oxygen species were enriched after treatment, indicating that biofilm formation was the main change in the first three months. Biotin is an essential nutrient belonging to the vitamin B complex and a coenzyme that is required for various critical biochemical processes, which include amino acid metabolism, gluconeogenesis, and fatty acid synthesis [38]. Bacterial biofilm formation depends on the assembly of extracellular matrix components (proteins, nucleic acids, polysaccharides, and other biomolecules) to facilitate the adhesion of cells. Vibrio polysaccharide (VPS) is a main constituent of the *V. cholerae* biofilm matrix present in mature biofilms. VPS facilitates the initial stages of microcolony and cluster formation through its interaction with matrix components, including biofilm matrix protein RbmA and extracellular DNA [39].

This study also focused on virulent factors and identified the genes associated with Type IV pili (VF0082), hemolysin (CVF417), flagella (VF0394), type IV pili (VF0075), and flagella (VF0430). Type IV pili (VF0082) and flagella (VF0394) were more abundant after treatment for three months. A previous study reported that type IV pili (VF0082) and flagella (VF0394) are offensive VFs and contribute to microorganism adhesion [40]. Various extracellular organelles, such as pili, flagella, capsules, and fimbriae control bacterial functions such as adhesion and motility. Similarly, the Type IV pili have retractile surface appendages, which facilitate several biological functions such as cell adhesion, surface-dependent twitching motility, and biofilm formation [41]. For example, the pili facilitate bacterial cell adhesion to the tooth surface by generating electrostatic adsorption in certain environments [42]. Furthermore, helical structures flagella establish adhesive interactions to facilitate the adhesion of both biotic and abiotic materials [42, 43]. Overall, the microbiome’s virulence factors at the early stage of CA therapy converted to offensive virulence factors conducive to microorganism adhesion and biofilm formation. Therefore, ensuring optimal oral hygiene during the initial phase of treatment is crucial for significantly enhancing the prevention of white spot lesions (WSLs). When designing clear aligners (CAs) with the goal of preventing caries in this initial phase, the primary emphasis should be placed on inhibiting bacterial adhesion and biofilm formation.

The present study has a few limitations, including its short duration and relatively small sample size. Therefore, enamel demineralization cannot be evaluated for a longer time. To address these limitations and investigate effects of CAs for a prolonged time, further research with bigger sample sizes and long-term observations is required to authenticate the results of the current research.

## Conclusions

To conclude, in adolescents undergoing CA therapy with poor plaque control, caries progress quickly within three months, and the individuals develop noticeable WSLs on the tooth surface. While the microbial community remained relatively steady and CA therapy did not cause significant changes in the overall functional gene composition in the first three months, virulence factors, including type IV pili and flagella, were more abundant and actively contributed to microorganism adhesion and biofilm formation.

## Data Availability

The datasets generated during and/or analyzed during the current study are available in the Sequence Read Archive repository (https://www.ncbi.nlm.nih.gov/sra/PRJNA1155131) under project No. PRJNA1155131.

## Abbreviations

WSLs: White spot lesions
CAs: Clear aligners
PI: Plaque index
ORFs: Open reading frames
PCoA: Principal coordinate analysis
NMDS: Nonmetric multidimensional
VFs: Virulence factors
VPS: Vibrio polysaccharide
